# Oxidative Stress and FOXO-1 Relationship in Stage III Periodontitis

**DOI:** 10.1007/s00784-024-05670-x

**Published:** 2024-04-25

**Authors:** Elif Selin Gurbuz, Zeliha Guney, Sivge Kurgan, Nur Balci, Muhittin Abdulkadir Serdar, Meral Gunhan

**Affiliations:** 1 Faculty of Dentistry Department of Periodontology, Ankara University, Ankara, Turkey; 2 Graduate School of Health Science, Ankara University, Ankara, Turkey; 3 Faculty of Dentistry Department of Periodontology, Ankara Medipol University, Ankara, Turkey; 4 Faculty of Dentistry Department of Periodontology, İstanbul Medipol University, Ankara, Turkey; 5 Faculty of Medicine, Department of Biochemistry, Acibadem University, Istanbul, Turkey

**Keywords:** **(MeSH):** Periodontitis, 8-Hidroksideoksiguanozin (8-OHdG), Forkhead Box-O1 (FOXO1), Oxidative Stress

## Abstract

**Objectives:**

8-Hydroxideoxyguanosine (8-OHdG) is a marker of oxidative stress, and Forkhead Box-O1 (FOXO1) is a transcription factor and signaling integrator in cell and tissue homeostasis. This study aims to determine FOXO1 and 8-OHdG levels in serum and saliva samples of periodontitis patients and to evaluate their relationship with clinical periodontal parameters.

**Materials and Methods:**

Twenty healthy individuals, twenty generalized Stage III Grade B periodontitis patients, and nineteen generalized Stage III Grade C periodontitis patients were included in the study. Clinical periodontal parameters (plaque index (PI), probing depth (PD), bleeding on probing (BOP), and clinical attachment level (CAL)) were recorded. Salivary and serum 8-OHdG and FOX-O1 levels were analyzed by enzyme-linked immunosorbent assay (ELISA).

**Results:**

Clinical periodontal parameters showed a statistically significant increase in periodontitis groups compared to the control group (p < 0.05). 8-OHdG salivary levels were significantly higher in both periodontitis groups compared to the control group. The salivary FOXO1 levels were significantly lower in both periodontitis groups compared to the control group. Salivary FOXO1 level had a low-grade negative correlation with BOP and salivary 8-OHdG level.

**Conclusions:**

While reactive oxygen species increase in periodontal inflammation, low expression of FOXO1, an important transcription factor for antioxidant enzymes, supports that this molecule plays a vital role in tissue destruction, and FOXO1 can be seen as a potential immune modulator.

**Clinical relevance:**

The role of FOXO1 in supporting antioxidant defense may suggest that FOXO1 is a candidate target for periodontitis treatment.

## Introduction

All organisms have various enzymatic and nonenzymatic antioxidant defense systems to protect themselves from the harmful effects of oxidative stress (OS). Under normal physiological conditions, there is a dynamic balance between reactive oxygen species (ROS) and antioxidant system (AO). When the balance disappears in favor of ROS, OS occurs with a decrease in antioxidant defense and/or an increase in ROS activity and causes varying degrees of damage to tissues [1, 2], such as depolymerization of extracellular matrix components, lipid peroxidation, oxidation of enzymes such as antiproteases, induction of proinflammatory cytokines and DNA damage [3, 4].

8-hydroxydeoxyguanosine (8-OHdG) is an oxidized nucleoside released by DNA damage. Studies have shown that 8-OHdG in body fluids acts as an oxidative stress marker and is used to evaluate oxidative damage in disorders, including chronic inflammatory diseases [5–8].

FOXOs are members of the O (other) class of the Forkhead superfamily called FKHR (forkhead rhabdomyosarcoma) [9]. Four members of this class are known as FOXO1, FOXO3, FOXO4, and FOXO6. While FOXO1, FOXO3, and FOXO4 play a role in apoptosis signaling [10, 11], FOXO1 and FOXO6 antagonize oxidative stress through the transcription of manganese superoxide dismutase, which catalyzes the conversion of O_2_^−^ to H_2_O_2_ [12, 13]. It helps cells maintain and counteract the effect of ROS by stimulating cell cycle inhibition [11, 14]. It has been observed that FOXO1 overexpression protects periodontal ligament cells against oxidative damage and increases their osteogenic capacity in the inflammatory setting [15].

As a result of studies examining the role of FOXO1 in antioxidant defense, it has been revealed that it is associated with some systemic inflammatory diseases such as cardiovascular diseases [16], brain diseases [17], and skeletal muscle diseases [18], but there is not enough data on its relationship with periodontitis. In this study, we tested the hypothesis that the FOXO1 may regulate antioxidant mechanisms in periodontal inflammation and aimed to evaluate the salivary and serum FOXO1 and 8-OHdG levels in advanced periodontitis patients.

## Materials and Methods

### Study Population

This cross-sectional study conducted at the Department of Periodontology, School of Dentistry, Ankara University, included a total of 39 systematically healthy non-smoker patients with generalized Stage III periodontitis (20 participants for the Grade B group and 19 participants for the Grade C group), and 20 systematically and periodontally healthy non-smoker individuals.

The study received ethical approval from the Human Subject's Ethics Board of Ankara University (Approval No: 03/10, dated 03.02.2021) and was conducted in compliance with the principles outlined in the Helsinki Declaration. Written consent was obtained from all participants before their involvement in the study.

The diagnosis was based on the 2017 World Workshop on the Classification of Periodontal and Peri-Implant Diseases and Conditions [19]. Periodontally healthy individuals were defined as having a probing depth (PD) ≤ 3 mm and no signs of inflammation. The diagnosis of Stage III periodontitis was made in patients exhibiting interdental radiographic bone loss of ≥ 2 mm in nonadjacent, distinct teeth, or buccal or oral radiographic bone loss up to 15% with a PD of > 3 mm for ≥ 2 teeth. The additional condition for Grade B was % bone loss/age 0.25–1, and for Grade C was % bone loss/age > 1 [19].

We excluded individuals from the study who had taken antibiotics, anti-inflammatory or immunosuppressive drugs, or contraceptives within the three months leading up to the research. Patients were excluded if they met any one of the following conditions: systemic disease (diabetes mellitus, rheumatoid arthritis, hypertension, metabolic syndrome, obesity), pregnancy, antibiotics, or anti-inflammatory medication for the last three months, active periodontal treatment in the previous six months, periodontal surgery in the last year, medications affecting the gingiva (e.g., calcium channel blocker, phenytoin, or cyclosporine). All participants were non-smokers, i.e., never smokers or had reportedly quit for a minimum period of 2 years before enrollment [20].

### Periodontal Clinical Parameters

All study participants were evaluated clinically on their first visit to the Departments of Periodontology by one trained and calibrated examiner (ESG). Full mouth periodontal examination included clinical measurements of probing depth (PD), clinical attachment level (CAL), and plaque index (PI) [21], recorded to the nearest mm, and bleeding on probing (BOP) (%) [22]. Clinical parameters were recorded at six tooth sites (mesio-buccal, mid-buccal, disto-buccal, mesio-lingual, mid-lingual, and disto-lingual).

### Saliva and Serum Sampling

Each patient was instructed on the collection protocol, and unstimulated saliva samples were obtained from them in the early hours of the day (between 9:00 am and 11:00 am). The participants were instructed to rinse their mouths with distilled water and sit comfortably while spitting into plastic tubes for 5 min. Subsequently, the saliva samples were centrifuged at 2800 × g for 10 min, and the resulting supernatant was transferred to Eppendorf tubes [7].

Standard venipuncture was performed for venous blood sample collection, and the samples were allowed to clot at room temperature for 30 min. Following clotting, the blood samples were centrifuged at 3000 × g for 10 min to separate the serum. All samples were stored at -80 °C until the day of analysis [7].

### 8-OHdG and FOX-O1 Analysis

The levels of 8-OHdG and FOX-O1 in serum and saliva samples were measured using commercially available ELISA kits.^1^ The assays were conducted following the instructions provided by the manufacturers, and colorimetric assessment was carried out using a microplate reader set at a wavelength of 450 nm^2^. The intra- and inter-assay accuracy for FOXO1 and 8-OHdG was 10% and 12%, respectively. The minimum detection ranges for the assays were as follows: FOXO1, 0.312-20 ng/mL; 8-OHdG, 74.07–6,000 pg/mL. Each sample was analyzed twice, and the average of the results was used for subsequent calculations.

### Statistical Analyses

Sample size analysis was performed a priori using specific software.^2^ Considering a large effect size (1) for the analysis involving three groups, an α-error of 0.05, and a power of 80%, the total sample size was 51 participants. However, considering the possibility of confounders and incomplete data, the study was designed to include 59 patients.

All analyses were performed using commercially available statistical software.^3^ Shapiro Wilk test was used to evaluate whether data were normally distributed. Chi-square test was applied for categorical variables. The t-test was used between the two independent groups.

The Bonferroni test was used as a "one-way ANOVA test" multiple comparison test to compare variables among the three groups. The Kruskal–Wallis test was used for data that were not normally distributed. Correlations between biochemical and periodontal clinical parameters were performed using Spearman correlation analysis. A value of p < 0.05 was considered significant.

## Results

### Study Population and Periodontal Clinical Parameters

Systemically healthy, twenty patients with Stage III Grade B periodontitis (SIIIGB, nine females, 11 males; 27–61 years old, mean 38.9 ± 10.17) and nineteen patients with generalized Stage III Grade C periodontitis (SIIIGC, 11 females, eight males; 19–41 years old, mean 27.53 ± 5.46), and twenty periodontally healthy subjects (C, 11 females and nine males; 23–49 years old, mean 30.75 ± 8.6) were included in the study.

Demographic and periodontal clinical parameters are presented in Table 1**.** Periodontitis group had significantly higher clinical periodontal parameters than the control group (p < 0.05). The mean age of the SIIIGB group was statistically significantly higher than the other two groups (p < 0.05), but there was no significant difference between the groups in terms of gender, body mass index, and saliva flow rate (p > 0.05).

**Table 1:** Comparison of clinical periodontal parameters and demographic data between the groups

Clinical Parameters	Control (n = 20)	SIIIGB (n = 20)	SIIIGC (n = 19)
Age (year)	30.75 ± 8.6	38.9 ± 10.17*	27.53 ± 5.46^γ^
Sex (n, F/M)	14/6	9/11	11/8
SFR ml/min	0.40 (0.22–0.48)	0.32 (0.22–0.47)	0.29 (0.20–0.46)
BMI	22.2 (20.8–24.9)	25.6 (21.5–28.5)	23.4 (22.4–25.6)
PI	0.80 (0.62–1.01)	1.67 (1.35–2.32)*	2.08 (1.96–2.16)*
PD (mm)	1.56 (1.45–1.64)	4.40 (3.69–5.15)*	5.93 (5.17–6.20)*
BOP (%)	3.50 (1.75–8.75)	56.70 (39.25–81.50)*	100 (100–100)*^γ^
CAL (mm)	0.00 (0.00–0.03)	4.68 (4.31–5.65)*	6.00 (5.60–6.76)*

SFR: Saliva Flow Rate; BMI: Body Mass Index, PI: plaque index; PD: probing depth; BOP: bleeding on probing; CAL: clinical attachment lost

Data are shown as mean ± standard deviation. Student-T test

Data are shown as median-interquartile range. Kruskal–Wallis test

^*^ Statistically significant difference compared to the control Group (p < 0.05)

^γ^ Statistically significant difference between the two disease groups (p < 0.05)

### Biochemical Parameters

**Fig. 1:**
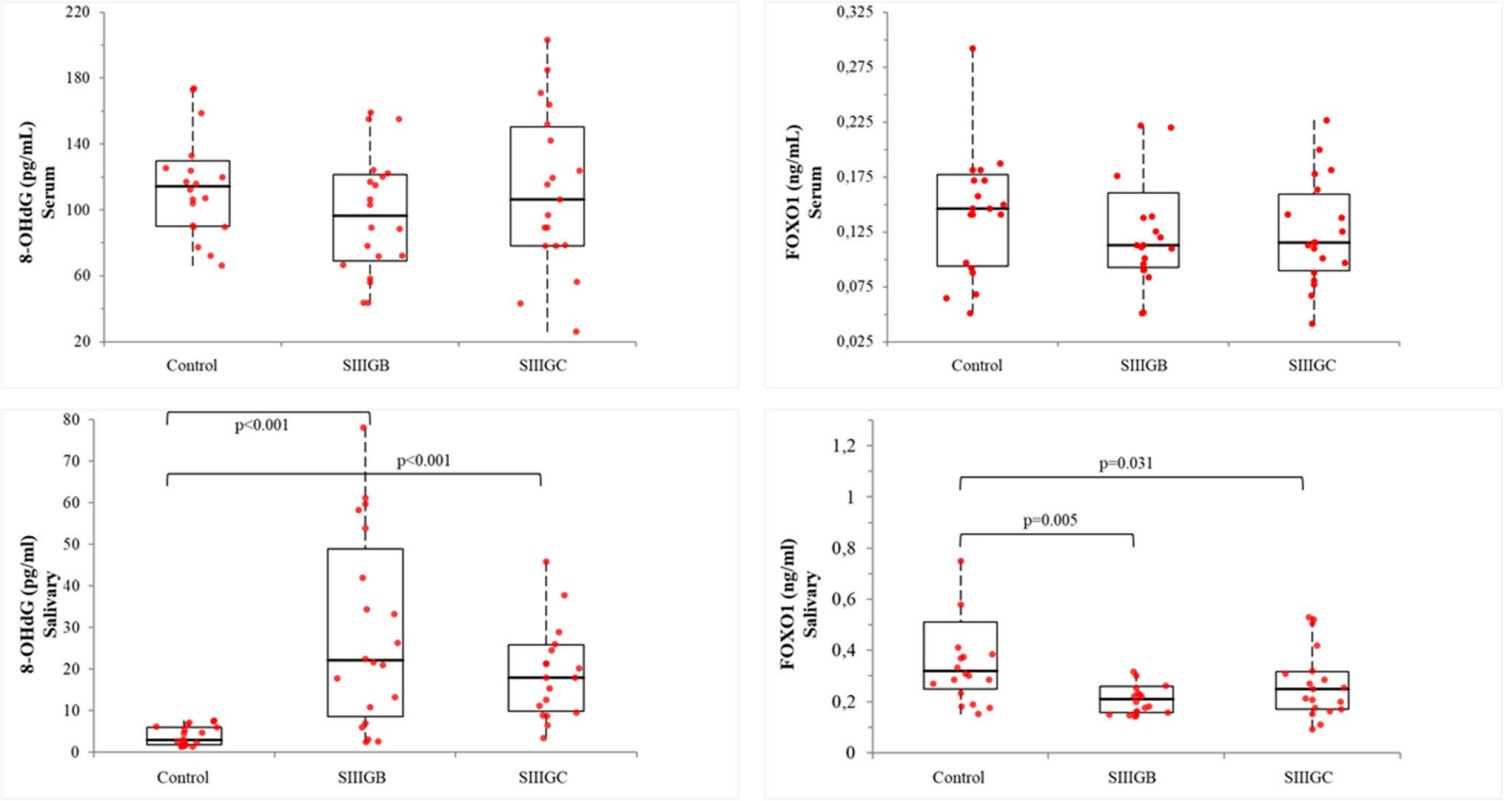
Salivary and serum levels of 8-OHdG and FOXO1

Salivary and serum 8-OHdG and FOXO1 levels are presented in Fig. 1. Salivary 8-OHdG levels were statistically significantly increased in the periodontitis groups compared to the control group (p < 0.05). Salivary FOXO1 levels were statistically significantly decreased in the periodontitis groups compared to the control group. However, no significant difference was found between the periodontitis groups in terms of 8-OHdG and FOXO1 levels (p > 0.05). Serum 8-OHdG and FOXO1 levels were similar in periodontitis and control groups (p > 0.05).

### Correlations Between Periodontal and Biochemical Parameters

The correlation between periodontal and biochemical parameters is shown in Table 2. Salivary 8-OHdG level was positively correlated with all clinical periodontal parameters (p and rho values are 0.570/0.001 for PD; 0.573/ < 0.001 for BOP; 0.470/0.00; 0.600/ < 0.001 for CAL) and negatively correlated with serum 8-OHdG level (-0.259/0.048). Salivary FOXO1 level was negatively correlated with BOP (-0.336/0.010) and salivary 8-OHdG level (0.395/0.002).

**Table 2:** Correlations between biomarkers and periodontal clinical parameters

Variables	FOXO1 (saliva)	8-OHdG (saliva)	FOXO1 (serum)	8-OHdG (serum)
	rho/p	rho/p	rho/p	rho/p
8-OHdG (saliva)	-0.395**/0.002**	**-**	-	**-**
FOXO1 (serum)	0.083/0.475	-0.143/0.280	**-**	-
8-OHdG (serum)	0.328**/0.013**	-0.259**/0.048**	-0.151/0.252	**-**
PD	-0.251/0.051	0.563**/ < 0.0001**	-0.110/0.359	-0.087/0.560
BOP	-0.333**/0.010**	0.573**/ < 0.0001**	-0.153/0.247	-0.091/0.493
PI	-0.201/0.115	0.470**/0.000**	-0.049/0.710	-0.093/0.484
CAL	-0.253/0.052	0.606**/ < 0.0001**	-0.125/0.386	-0.034/0.730

8-OHdG: 8-hydroxydeoxyguanosine; FOXO1: Forkhead Box-O1

Values in bold are different from 0 with a significance level of alpha < 0.05, Spearmen correlation test

## Discussion

This study aimed to evaluate the salivary and serum FOXO1 and 8-OHdG levels in Stage III periodontitis patients. Based on the hypothesis that FOXO1 may regulate antioxidant mechanisms in periodontal inflammation, we evaluated and compared saliva and serum samples obtained from SIIIGB and SIIIGC with non-periodontitis healthy controls. While salivary 8-OHdG level was statistically significantly higher in periodontitis groups, salivary FOXO-1 level was significantly lower.

Recent studies have shown a significant relationship between periodontal disease and oxidative stress [23–29], and 8-OHdG is accepted as a sensitive indicator of DNA damage and a marker that defines oxidative stress [30, 31]. As a result of this study, salivary 8-OHdG levels were significantly higher in periodontitis groups than in the control group, consistent with the literature [32–34]. On the other hand, salivary 8-OHdG levels did not show significant difference between the periodontitis groups. Considering the literature data, 8-0HdG levels are accepted as a marker of periodontal inflammation [35, 36]. According to the 2017 World Workshop on the Classification of Periodontal and Peri-Implant Diseases and Conditions [19], Stage III periodontitis is graded according to bone loss/age, and subclassification in our periodontitis group is made accordingly. It was evaluated whether there was a difference between these grades regarding oxidative stress and FOXO. As a result of this evaluation, no difference was found between Grade B and Grade C.

Although there is a difference in salivary 8-OHdG levels between the periodontitis and control groups, serum 8-OHdG levels were similar in periodontitis and control groups. Similarly, Konopka et al. observed no difference in serum 8-OHDg levels between periodontitis and control groups [37]. The researchers concluded that the oxidative burst observed in periodontitis is extensive enough to cause significant DNA damage at the local level. The level of 8-OHdG can be higher than the plasma concentration in the microcirculation and effectively change the redox status locally.

FOXO1, a transcription factor, plays a crucial role in regulating various cellular processes such as cell survival, differentiation, the reduction of ROS, and apoptosis [38, 39]. In the current study, salivary FOXO1 levels were statistically significantly lower in periodontitis groups than in the control group. Antioxidant systems are expected to be activated to prevent or reduce tissue damage caused by oxidative stress in periodontal disease. Accordingly, FOXO1 levels, whose antioxidant effect is known, could be expected to be higher in periodontitis groups. However, it can be thought that the high level of oxidative stress (8-OHdG) may prevent the functioning of antioxidant systems [40]. However, it is also known that ROS regulates the localization and activation of FOXO1. In case of an increase in OS, it causes FOXO1 to be inactivated by its acetylation by being transported out of the nucleus and reduces target gene expression [41]. On the other hand, it may be another interpretation to assume that the destruction is severe due to the insufficient functioning of the antioxidant mechanism in the periodontal destruction process. On the contrary, when activated, FOXO1 functions as a vital signal integrator, contributing to maintaining homeostasis and adapting to environmental shifts [42], which are crucial for periodontal health. Consequently, Ren et al. highlighted that disturbances in the normal signaling of FOXO1 could have implications for periodontal dysbiosis [43]. From this point of view, we can also consider that FOXO1 levels were lower than the control group in our study as the deterioration of physiological FOXO1 signal in periodontal inflammation. Interestingly, despite growing evidence indicating that periodontal pathogens can induce FOXO1 activity [43, 44] to maintain its intracellular existence by inhibiting apoptosis [44], the specific roles of FOXO1 in periodontal homeostasis and disease are not extensively documented.

FOXO1 plays a crucial role in regulating different aspects of mucosal immunity by influencing the migration and activation of dendritic cells, macrophages, and neutrophils. It also impacts the development and function of T-helper cells and B-lymphocytes [45–47]. Moreover, FOXO1 controls cytokine production, protects hematopoietic stem cells from oxidative stress, and regulates vital functions of keratinocytes, potentially contributing to the maintenance or restoration of the epithelial barrier [48, 49]. However, the effects and role of FOXO1 can vary depending on the specific conditions, such as diabetes [50, 51]. Consequently, predicting the impact of FOXO1 on various diseases can be challenging. In subsequent studies, examining the behavior of FOXO1 in different stages of periodontitis will highlight the intricate nature of FOXO. These findings indicate that epigenetic factors like high glucose levels or elevated oxidative stress highly regulate it.

The cross-sectional nature of this study is its main limitation; due to the study design, randomization in the selection of patients was not possible.

Our results showed a negative correlation between salivary 8-OHdG and FOXO1 levels. It is known that oxidative stress is one of the most critical factors in the tissue destruction mechanism in periodontal breakdown. In contrast, a deficiency in all antioxidant systems in the disease process is mentioned [52]. Considering all these, the negative correlation of FOXO1 saliva level with 8-OHdG may also be related to the nature of the disease.

Gaining a deeper understanding of the mechanisms involved in protection against oxidative stress, which is an essential factor in the development of periodontal disease, will be valuable for a more comprehensive understanding of the pathology of periodontitis. Literature data indicate that FOXO-1 may be a new therapeutic agent in some inflammatory diseases such as diabetes [53], obesity [54], and hypertension [55]. However, no study confirms FOXO1 as a therapeutic agent in periodontal disease. More in vitro and in vivo studies are needed to ensure the role of FOXO1 in promoting tissue regeneration in periodontitis, the immune system, and osteogenesis.

## Conclusions

According to our results, the oxidative stress (8-OHdG)/antioxidant (FOXO1) ratio in patients with periodontitis varies in favor of oxidative stress compared to the healthy group. FOXO1 is an essential parameter of the antioxidant system that plays a role in the pathogenesis of periodontal disease, and this role should be elucidated in further studies.

## Footnotes

1 ELISA Cloud Immunoassay, Cloud-Clone Corp, Houston, USA.

2 3.0.10 G*Power; https://www.psychologie.hhu.de/arbeitsgruppen/allgemeine-psychologie-und-arbeitspsychologie/gpower.html

3 SPSS for Windows R4.
